# Response to Pairojkul et al. Integration of Specialist Palliative Care into Tertiary Hospitals: A Multicenter Point Prevalence Survey from Thailand (DOI: 10.1089/pmr.2021.0003)

**DOI:** 10.1089/pmr.2022.0001

**Published:** 2022-02-08

**Authors:** Rujittika Mungmunpuntipatip, Viroj Wiwanitkit

**Affiliations:** ^1^ Private Academic Consultant, Bangkok Thailand.; ^2^ Honorary Professor, Dr DY Patil University, Pune, India.

*Dear Editor:*

We would like to comment on “Integration of Specialist Palliative Care in Tertiary Hospitals: A Multicenter Point Prevalence Survey from Thailand.”^1^

Pairojkul et al. noted that “The prevalence of hospitalized palliative care patients in Thailand is comparable with that in developed countries; however, accessibility remains a significant gap, as specialist palliative care is associated with the quality of palliative care service.” The authors noted that data came from tertiary care centers in different regions of a developing country and included data from several provincial and district hospitals. In Thailand, district hospitals are considered primary care centers and provincial hospitals are secondary centers. We worry that the nonhomogeneous sources of data might affect overall features of reported incidence.

In addition, the “Supportive and Palliative Care Indicators Tool (SPICT(TM)) version 2017” was employed. The reliability of the tool, however, is not clear for a non-English-speaking sample. We would encourage data verification on a modified tool when translated into another language.^2^
